# Targeting cancer and immune cell metabolism with the complex I inhibitors metformin and IACS‐010759

**DOI:** 10.1002/1878-0261.13583

**Published:** 2024-01-12

**Authors:** Marc Pujalte‐Martin, Amine Belaïd, Simon Bost, Michel Kahi, Pascal Peraldi, Matthieu Rouleau, Nathalie M. Mazure, Frédéric Bost

**Affiliations:** ^1^ Inserm U1065, Centre Méditerranéen de Médecine Moléculaire (C3M) Nice France; ^2^ Equipe Labellisée Ligue Nationale Contre le Cancer; ^3^ Faculté de Médecine Université Côte d'Azur Nice France; ^4^ CNRS UMR7370, LP2M Nice France

**Keywords:** cancer, clinical trial, IACS‐010759, immunometabolism, metformin, microenvironment

## Abstract

Metformin and IACS‐010759 are two distinct antimetabolic agents. Metformin, an established antidiabetic drug, mildly inhibits mitochondrial complex I, while IACS‐010759 is a new potent mitochondrial complex I inhibitor. Mitochondria is pivotal in the energy metabolism of cells by providing adenosine triphosphate through oxidative phosphorylation (OXPHOS). Hence, mitochondrial metabolism and OXPHOS become a vulnerability when targeted in cancer cells. Both drugs have promising antitumoral effects in diverse cancers, supported by preclinical *in vitro* and *in vivo* studies. We present evidence of their direct impact on cancer cells and their immunomodulatory effects. In clinical studies, while observational epidemiologic studies on metformin were encouraging, actual trial results were not as expected. However, IACS‐01075 exhibited major adverse effects, thereby causing a metabolic shift to glycolysis and elevated lactic acid concentrations. Therefore, the future outlook for these two drugs depends on preventive clinical trials for metformin and investigations into the plausible toxic effects on normal cells for IACS‐01075.

## Graphical abstract

Metformin, an established antidiabetic drug, mildly inhibits mitochondrial complex I, while IACS‐010759 is a potent complex I inhibitor. Both target cancer cells' vulnerability by impacting mitochondrial metabolism and oxidative phosphorylation. Promising preclinical results support their antitumoral effects. Clinical studies show metformin's mixed outcomes and IACS‐01075's adverse effects. Prospects hinge on preventive metformin trials and investigations into IACS‐01075's toxic effects on normal cells.

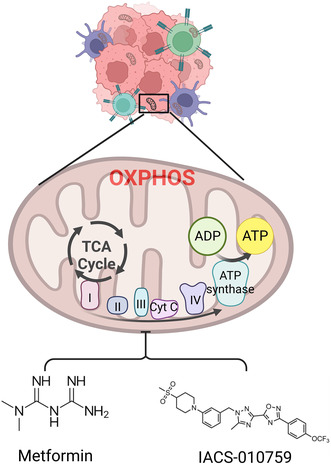

**Abbreviations**
ADP: adenosine diphosphate
AMPK: AMP‐activated protein kinase
ATP: adenosine triphosphate
DC: dendritic cells
DLBCL: diffuse large B‐cell lymphoma
ETC: electron transport chain
FAO: fatty acid oxidation
HIF: hypoxia‐inducible factor
LDH: lactate dehydrogenase
ND1: NADH dehydrogenase 1
NRF2: nuclear factor erythroid 2‐related factor 2
OS: overall survival
OXPHOS: oxidative phosphorylation
PFS: progression‐free survival
TAM: tumor‐associated macrophages
TAN: tumor‐associated neutrophils
TCA: tricarboxylic acid cycle
TME: tumor microenvironment

## 1 Introduction

Since Warburg's pioneering work, it has been well‐established that cancer cells exhibit distinct metabolism compared with normal cells [1]. Warburg initially proposed that cancer cells preferentially rely on glycolysis, even in the presence of oxygen (aerobic glycolysis), to synthetize adenosine triphosphate (ATP). He also believed that cancer cells had dysfunctional mitochondria. However, current understanding affirms that cancer cells have functional mitochondria, utilizing them as a primary energy source and adapting their metabolism to changes in the tumor microenvironment (TME). In response to low oxygen (hypoxia), limited nutrient availability, low pH, and mechanic cues, cancer cells undergo metabolic reprogramming [2], a concept recently refined by Fendt et al. [3] as metabolic flexibility (the ability to use different nutrients) and metabolic plasticity (the ability to process the same nutrients in different ways).

For the energy‐intensive processes like proliferation and invasion, cancer cells require active synthesis of proteins, lipids, and nucleotides, providing energy in the form of ATP, cofactors such as NADH, and other metabolites. Mitochondria play a crucial role in providing ATP through oxidative phosphorylation (OXPHOS), making mitochondrial metabolism and OXPHOS a vulnerability for cancer cells. However, tumors are not just masses of cancer cells; they represent dynamic ecosystems where immune and nonimmune cells interact intricately [4, 5]. The composition and behavior of immune infiltrates vary widely across different cancer types and even within the same tumor. Furthermore, immune cells significantly influence tumor progression and treatment resistance [6].

In recent years, two drugs inhibiting mitochondrial metabolism have attracted attention: metformin and IACS‐010759. Both target the complex I of the mitochondrial respiratory chain, with one being an established antidiabetic drug [7], and the other designed to exploit OXPHOS vulnerability [8]. This review, following a brief recap of the role of mitochondria in metabolic plasticity/flexibility and the metabolic characteristics of immune cells, provides an overview of the effects of metformin and IACS‐010759 on cancer and immune cells in both experimental and clinical studies.

## 2 Mitochondria empower cells to convert nutrients into energy

**Fig. 1:**
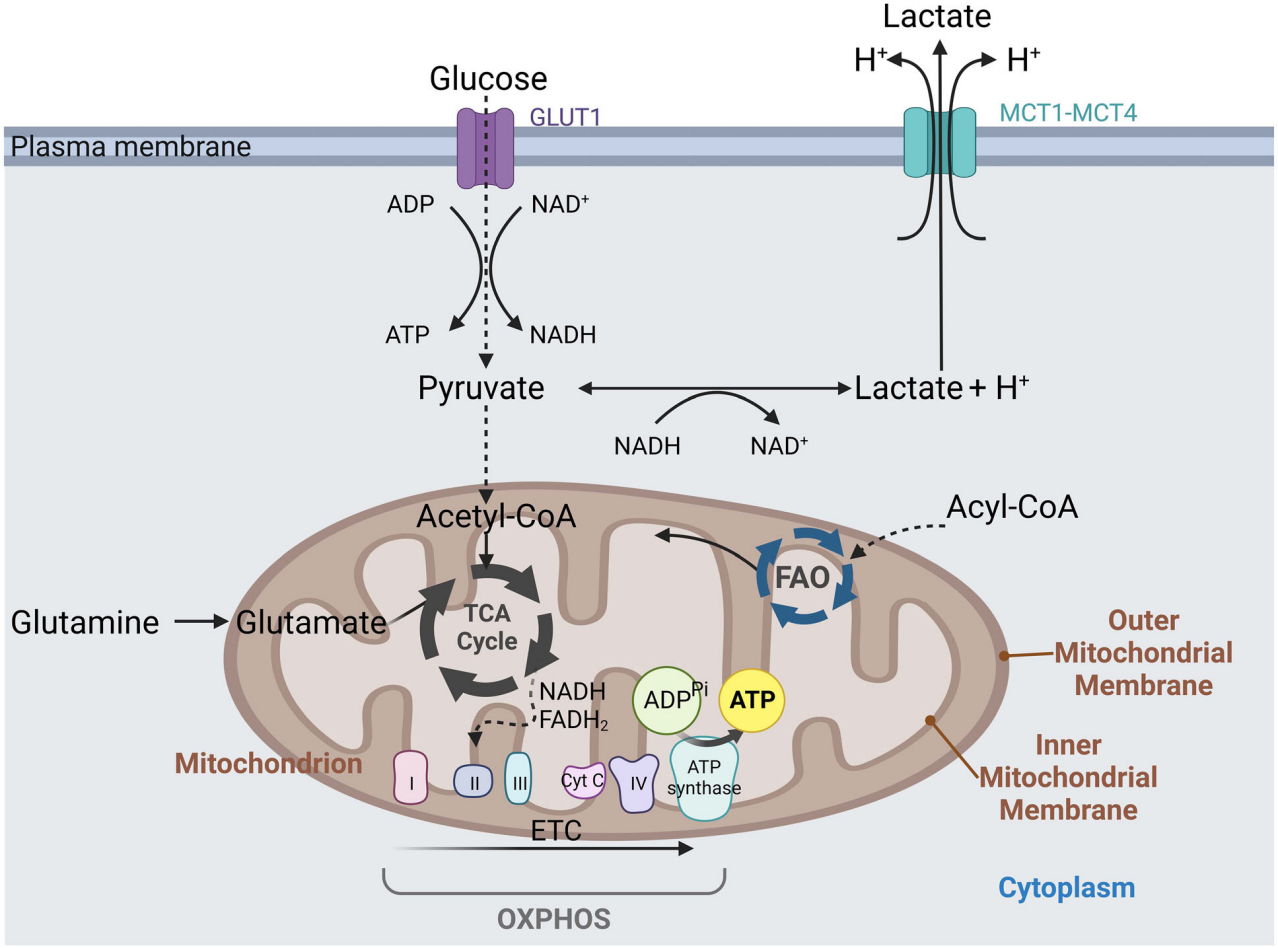
Mitochondrion at the center of cell metabolism. In cancer cells, glycolysis is the first step in the energy‐producing process: it converts glucose, which has entered the cell via glucose transporters (GLUTs), into pyruvate. For some cancer cells, regulation of oxidative phosphorylation (OXPHOS) and pyruvate utilization in the tricarboxylic acid (TCA) cycle plays a crucial role in their unique metabolic adaptations. Pyruvate is imported into the mitochondria, where it is converted to acetyl‐CoA. It then enters the TCA cycle, fueling a series of metabolic reactions that generate reducing agents such as NADH and FADH2. The electron transport chain (ETC) is a critical component of cellular respiration. The ETC is in the inner mitochondrial membrane and consists of a series of protein complexes (I to IV) and electron carriers, including coenzyme Q and cytochrome c. During cellular respiration, the ETC transfers electrons derived from metabolic reactions, such as glycolysis and the citric acid cycle, from one protein complex to another. This flow of electrons creates an electron gradient, which drives the pumping of protons (H^+^) across the inner mitochondrial membrane from the matrix to the intermembrane space. However, most cancer cells will convert pyruvate into lactate (Warburg effect) irrespective of the oxygen concentration of the microenvironment. Lactate will then be released outside the cell via monocarboxylate transporters (MCTs) simultaneously with protons (H^+^). Cancer cells have a heightened dependence on glutamine, an essential amino acid, to support their rapid growth and proliferation. Glutaminolysis involves the conversion of glutamine into glutamate by the enzyme glutaminase. Glutamate can then enter the TCA cycle as α‐ketoglutarate, providing intermediates for the synthesis of lipids, nucleotides, and nonessential amino acids required for cell growth and proliferation. They can also utilize fatty acid oxidation (FAO) as a source of energy, particularly in nutrient‐poor environments. FAO involves the breakdown of fatty acids into acetyl‐CoA, which enters the citric acid cycle to generate adenosine triphosphate (ATP). (Figure done with BioRender).

The maintenance of mitochondrial function is essential for cell survival and proliferation. These double‐membraned organelles not only play a significant role in apoptosis through the activation of the intrinsic pathway and transiently store calcium for calcium homeostasis (see review [9]). But mitochondria are commonly referred to as ‘powerhouses’ of the cell due to their vital role in energy production, specifically ATP synthesis [10]. The electron transport chain (ETC) and ATP synthase complexes within mitochondria facilitate OXPHOS, where electrons flow through protein complexes I–IV (Fig. 1), creating a flow of electrons with oxygen being the last electron acceptor. This flux drives the pumping of protons from the mitochondrial matrix into the intermembrane space, which creates an electrochemical gradient across the inner mitochondrial membrane. The flow of protons back into the matrix through ATP synthase, located in the inner membrane, drives the synthesis of ATP from adenosine diphosphate (ADP) and inorganic phosphate (Pi) [11].

The interplay between nutrient availability and oxygen levels influences cancer initiation, tumor growth, and metastasis formation [12]. Beyond glycolysis, mitochondria play a pivotal role in converting nutrients into energy through OXPHOS. Glutamine, a principal substrate, enters the citric acid cycle, contributing to OXPHOS and ATP generation [13]. Proliferating tumor cells exhibit metabolic flexibility, utilizing both glucose and glutamine to meet their energetic and biosynthetic demands. Additionally, mitochondria actively participate in fatty acid metabolism, contributing to energy production and lipid synthesis essential for cellular membranes and signaling molecules [14]. Fatty acids undergo beta‐oxidation within mitochondria, generating acetyl‐CoA, a key precursor for the tricarboxylic acid (TCA) cycle. This integration of fatty acid metabolism further underscores the multifaceted role of mitochondria in sustaining anabolic requirements of tumor cells.

In the TME, varying oxygen levels lead to distinct metabolic and cellular responses. Under physioxic conditions, cancer cells utilize both OXPHOS and glycolysis for ATP generation [15]. In contrast, hypoxia creates challenges, triggering the activation of hypoxia‐inducible factor (HIF), the key orchestrator of the adaptative response to hypoxia, and exacerbating glycolysis [16, 17]. Hypoxia also shapes the TME, attracting immunosuppressive cells and hindering antitumor immune responses [18].

In conclusion, understanding tumor adaptation to nutrient/oxygen availability is a subject of extensive, offering potential insights into new therapeutic strategies. However, translating our understanding of tumor nutrient dependency into patient‐relevant interventions remains challenging.

## 3 Immune cell fate is influenced by energy metabolism

The important concepts of immune cell metabolism and their ability to adapt to various stresses in the context of cancer have been extensively addressed in numerous reviews [19, 20, 21, 22, 23, 24, 25, 26]; hence, these topics will not be discussed here.

The immune system intricately navigates a dual role in cancer, characterized by proinflammatory responses and contributions to wound‐healing and protumorigenesis. On the one hand, the immune system is equipped to recognize and eliminate cancer cells, employing T cells and natural killer cells in an antitumor immune response. This proinflammatory environment involves the release of cytokines, chemokines, and the activation of cytotoxic immune cells. On the other hand, the TME often adopts a wound‐healing phenotype, fostering chronic inflammation and immune suppression. Tumor‐associated macrophages (TAMs), tumor‐associated neutrophils (TANs), and other myeloid immunosuppressive cells contribute to tissue repair and angiogenesis but can also support tumor growth. Nutrient and oxygen availability further shape this dynamic. Rapidly growing cancer cells outcompete immune cells for resources, creating a nutrient‐deprived environment that hampers immune function. Hypoxia within the tumor, induced by insufficient oxygen supply, alters immune cell functions, and promotes an immunosuppressive microenvironment. The interplay between the immune system, nutrient availability, and oxygen levels is pivotal in determining the outcome of the immune response in cancer.

## 4 Metformin targets energy metabolism in cancer cells and immune cells

In the 2000s, metformin emerged as a promising candidate for targeting mitochondrial metabolism. Known for its inhibition of mitochondrial metabolism [27] and activation of AMP‐activated protein kinase (AMPK), the energy sensor of cells [28], metformin belongs to the biguanide family and was initially isolated from the plant *Galega officinalis* [29]. It has been used for more than 50 years by millions of type II diabetic patients, it is a safe drug with minimal side effects, such as rare instances of lactic acidosis (1/10 000 patients) and bowel upset (15% of patients) [30].

Retrospective epidemiological studies have demonstrated a decrease in cancer incidence among diabetic patients treated with metformin (see below). *In vitro* and *in vivo* have shown that metformin inhibits cancer cell growth, metastasis, and tumor growth across various cancers, including prostate, breast, lung, liver, glioblastoma, colon, ovary, and others [31, 32]. Mechanistically, metformin inhibits mitochondrial metabolism, leading to a decrease in ATP concentration [33, 34]. Pioneering work of El‐Mir et al. [27] in hepatocytes exhibited metformin's inhibition of complex I of the ETC, a finding later confirmed in cancer cells by our laboratory [34]. In response to the decreased ATP production, cancer cells undergo metabolic reprogramming, increasing glucose uptake, and lactate production [34]. Fendt et al. [35] observed that metformin enhances glutaminolysis, replenishing the TCA cycle through the conversion of glutamate into alpha‐ketoglutarate. Similarly, an increase in serine, a precursor of nucleotides and lipids, was observed in response to metformin in nonsmall cell lung cancer and colon cancer cells [36]. Thus, strategies targeting these metabolic adaptations (i.e., increased glucose, glutamine, and serine uptake) have been explored to enhance the effectiveness of metformin. For instance, the combination of 2‐deoxyglucose, an inhibitor of glucose metabolism, with metformin induces significant energetic stress and apoptosis in prostate cancer cells. Metformin alone, however, arrests the cell cycle in G0/G1 without affecting cell survival [34, 37]. In colon cancer, a lactate dehydrogenase (LDH) inhibitor, oxamate, synergizes with phenformin, another biguanide, by inducing apoptosis and reducing tumor growth [38]. Additionally, combination therapies using metformin and compound 968 or CB‐839 that block glutaminase, the enzyme that converts glutamine into glutamate, significantly reduced tumor progression in prostate cancer [35] and osteosarcoma [39]. Similarly, the combination of metformin and l‐asparaginase, which hydrolyzes l‐asparagine, a source of glutamate, induced a complete response in patients with diffuse large B‐cell lymphoma (DLBCL) [40]. Finally, serine deprivation in cell culture medium and mouse diet potentiated metformin's antineoplastic activity [36].

The question of whether the inhibition of complex explains a potential antitumoral effect remains unclear. Wheaton et al. [41] demonstrated that the antitumoral effects of metformin were directly linked to complex I inhibition. They overexpressed the *Saccharomyces cerevisiae* NADH dehydrogenase (ND1), which oxidizes NADH in a process similar to the multi‐subunit mammalian complex I and showed that the antiproliferative effects of metformin were reversed in cells expressing ND1.

Contrarily, other studies have shown that the antiproliferative effects of metformin are independent of complex I inhibition. For example, Blandino et al. [42] implicated mir33a and c‐Myc in the anticancer effects of metformin in breast cancer. More recently, Kurelac et al. [43] demonstrated that metformin decreases proliferation and induces apoptosis in cancer cells devoid of complex I. Complicating matters, the concentration of metformin used to inhibit complex I *in vitro* (millimolar) differs from the micromolar concentrations observed in patient plasma [44]. A recent study also showed metformin's ability to ameliorate the dysfunctional mitochondrial function in peripheral blood mononuclear cells (PBMCs) of patients with type 2 diabetes [45]. The beneficial impact of metformin was attributed to the restoration of mitophagy and increased mitochondrial biogenesis. These contradictory results highlight the complexity of metformin's action in cancer and normal cells as illustrated in type 2 diabetes for which the antihyperglycemic action is not fully understood [30].

**Fig. 2:**
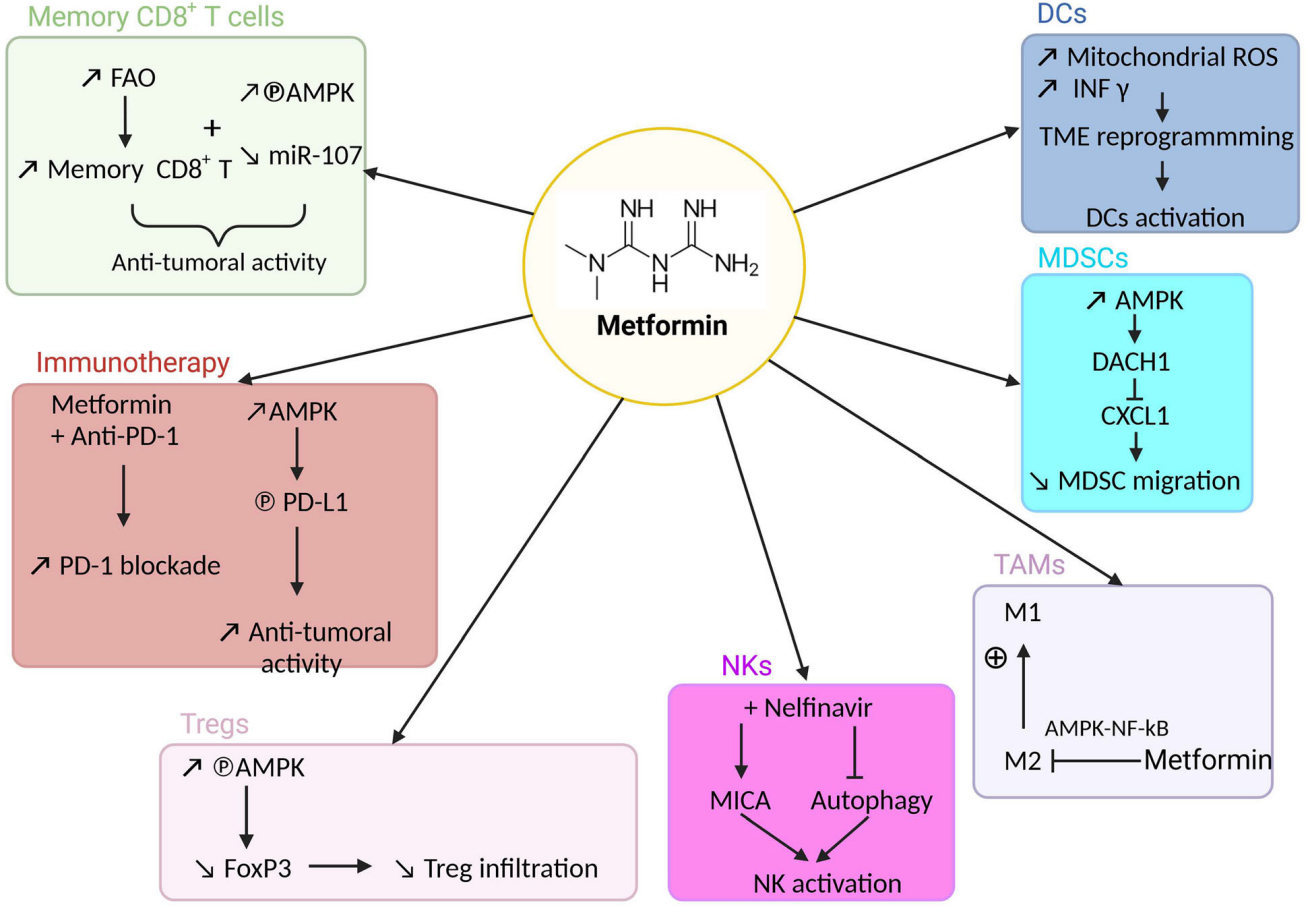
Metformin and immune response. Comprehensive overview of the relationship between metformin and the immune system. This illustration encapsulates the multifaceted effects of metformin on immune cells, highlighting its potential to modulate memory CD8^+^ T cells, immunotherapy, regulatory T cells (Tregs), natural killer cells (NKs), tumor‐associated macrophages (TAMs), myeloid‐derived suppressor cells (MDSCs) and dendritic cells (DCs). (Figure done with Biorender).

Emerging research shows that metformin displays immunomodulatory effects, which have been recently reviewed by Kurelac et al. [46]. Pearce et al. [47] were among the first to demonstrate the benefits of metformin on immune cells. They showed that metformin increased the number of memory CD8^+^ T cells through the activation of fatty acid oxidation (FAO) in T cells (Fig. 2). Several other reports have shown that targeting OXPHOS through metformin treatment increases the quantity and quality of CD8^+^ T cells in the context of obesity, antibacterial response, and cancer [48, 49]. Recently, Finisguerra et al. [50] observed an enhancement in the infiltration of CD8^+^ T cells in the presence of metformin, particularly under hypoxic conditions.

Tregs are obvious targets of metformin, given their reliance on OXPHOS for survival. Several studies have shown that metformin reduces Treg infiltration in the TME [51, 52, 53]. On the contrary, metformin enhances the antitumor properties of NK cells [54, 55]. It promotes NK cell toxicity against various cancer cell lines (melanoma, lung, and breast) both *in vitro* and *in vivo* through the p38MAPK pathway and strengthens the therapeutic efficacy of anti‐PD‐1 against melanoma [56]. A recent study further showed that metformin increased NK cell cytotoxicity in patients with head and neck cancer, promoting NK cell infiltration in tumors and perforin release [57].

In the context of cancer, most studies report an inhibitory role of metformin on M2 macrophages (pro‐tumorigenic; reviewed in [46, 58]). However, in conditions such as obesity [59, 60], atherosclerosis [61], and bone‐associated pathologies [62] metformin induces M2 polarization.

Immunotherapy based on PD‐1/PD‐L1 inhibition has demonstrated clinical efficacy in treating several cancers. For instance, in mouse models, the combination of metformin and anti‐PD‐1 reduces tumor growth compared with anti‐PD‐1 alone [63]. Metformin induces metabolic reprogramming by reducing oxygen consumption, thereby improving hypoxia and PD‐1 blockade. However, this finding is controversial, as the inhibition of OXPHOS increases lactate production, a condition supporting immunosuppression in the TME. Cha et al. [64] demonstrated that AMPK activated by metformin directly phosphorylates PD‐L1, inducing endoplasmic deregulation and potentiating the antitumoral activity of cytotoxic T lymphocytes. More recently, these results were confirmed *in vivo* in triple‐negative breast cancer xenografts with the combination of metformin or rapamycin (inhibitors of mTORC1) and anti‐PD‐1 antibodies [65].

In conclusion, the relationship between metformin and the immune response is a complex and evolving field of research with intriguing potential implications. A recent paper by Rodriguez group showed that a metformin dimer, called supformin, interferes with human immune cell plasticity, including dendritic cells (DCs) and macrophages. These results could have significant applications in the regulating cancer progression [66], and holding promise for the development of innovative therapeutic approaches.

## 5 From observational epidemiologic studies to clinical trials with metformin

Numerous observational studies across various cancer types have shown a protective effect of metformin against cancer in diabetic patients (see Review [31]). Meta‐analyses have substantiated these findings. For instance, concerning prostate cancer, a meta‐analysis involving 1 660 795 patients from 30 studies analyzed prostate cancer incidence, overall survival (OS), cancer‐specific survival, and recurrence‐free survival. The outcomes were very clear: metformin therapy exhibited improvement in incidence (HR = 0.72 [95% CI: 0.59–0.88]), OS (HR = 0.44 [95% CI: 0.35–0.55]), cancer‐specific survival (HR = 0.18 [95% CI: 0.07–0.45]), and relapse‐free survival (HR = 0.41 [95% CI: 0.39–0.58]) compared with patients not taking metformin [67].

In an observational study of 44 541 women with breast cancer, type 2 diabetes correlated with a 40% increased risk compared with nondiabetic women [68]. Metformin use, when compared to no type 2 diabetes, demonstrated a 24% decreased risk of estrogen receptor‐positive breast cancer and a 25% increased risk of estrogen receptor‐negative breast cancer. However, among the 44 541 individuals in the study, only 277 had breast cancer in the type 2 diabetes population, including 25 with triple‐negative breast cancer. The phase III ALTTO trial randomized 8000 patients with HER2^+^ primary breast cancer to different treatment options, such as trastuzumab, lapatinib, their sequence, or their combination. Retrospective analysis of this study suggested that metformin might improve the poorer prognosis of breast cancer in diabetic patients, aligning outcomes with nondiabetic population [69].

Based on these observational results and encouraging preclinical studies, numerous prospective studies have been conducted to evaluate the antitumoral effects of metformin, with over 400 clinical trials listed on ClinicalTrials.gov. While several trials are ongoing, only a limited number have published results to date, with the most significant ones reviewed below; a comprehensive list is provided in Table 1.

**Table 1:** List and outcomes of metformin clinical trials. ADT, androgen deprivation therapy; CRPC, castration‐resistant prostate cancer; DFS, disease‐free survivals; EGFR‐TKI, tyrosine kinase inhibitors of epidermal growth factor receptor activating mutation; mPFS, median progression‐free survival; NSCLC, nonsmall cell lung cancer; ORR, objective response rate; OS, overall survival; PEXG, cisplatin, epirubicin, capecitabine, gemcitabine; PFS, progression‐free survival; SOC, standard of care.

Cancer type	Study	Population	Treatments	*N*	Study design	Outcomes of the primary endpoint	Positive/negative study
Prostate cancer	Rothermundt et al. (2014) [122]	Advanced/metastatic CRPC No prior chemotherapy Nondiabetic	ADT continuation + Metformin	44	NA	Disease progression at 12 weeks: 9.1%	NA (no randomization)
	Alghandour et al. (2021) [74]	High‐risk localized OR advanced/metastatic hormone‐sensitive prostate cancer Diabetic and nondiabetic	SOC ± Metformin	124	Phase 2	Median Castration‐resistant prostate cancer‐free survival: 29 vs. 20 months (*P* = 0.01)	Positive
	Pujalte Martin et al. (2021) [73]	Advanced/metastatic CRPC No prior chemotherapy Nondiabetic	Docetaxel ± Metformin	99	Phase 2	PSA‐response rate: 66% vs. 63% (*P* = 0.94)	Negative
Lung cancer	Sayed et al. (2015) [123]	Advanced/metastatic NSCLC in first‐line setting Nondiabetic	Cisplatin + gemcitabine ± Metformin	80	Phase 2	ORR: 46.7% vs. 13.3% (*P* = 0.109)	Negative
	Marrone et al. (2018) [124]	Advanced/metastatic NSCLC Nondiabetic	Carboplatin + Paclitaxel + Bevacizumab ± Metformin	25	NA	1‐year PFS: 47% with metformin, so few patients without metformin	Negative
	Arrieta et al. (2019) [81]	Advanced/metastatic NSCLC with an activating EGFR mutation Nondiabetic	EGFR‐TKIs ± Metformin	139	Phase 2	mPFS: 13.1 vs. 9.9 months, HR = 0.60, 95% CI [0.40–0.94] (*P* = 0.01)	Positive
	Li et al. (2019) [125]	Advanced/metastatic NSCLC with an activating EGFR mutation. Nondiabetic	Gefetinib ± Metformin	224	Phase 2	PFS at 1 year: 41.2% vs. 42.9% (*P* = 0.63)	Negative
	Lee Y. Lung et al. (2021) [126]	Advanced/metastatic NSCLC in first‐line setting Diabetic and nondiabetic	Carboplatin + Gemcitabine ± Metformin	164	Phase 2	mPFS: 4.6 vs. 4.3 months HR = 1.01, 95% CI [0.72–1.42] (*P* = 0.935)	Negative
	Skinner et al. (2021) [79]	Stage III NSCLC Nondiabetic	Concurrent chemoradiotherapy followed by two cycles of chemotherapy ± Metformin	167	Phase 2	1‐year PFS: 51.3% vs. 60.4% (*P* = 0.24)	Negative
	Tsakiridis et al. (2021) [80]	Stage III NSCLC Nondiabetic	Concurrent chemoradiotherapy followed by two cycles of chemotherapy ± Metformin	117	Phase 2	1‐year PFS: 34.8% vs. 63.0%, HR = 2.42, 95% CI [1.14–5.10]	Negative
Breast cancer	El Haggar et al. (2016) [127]	In adjuvant BC setting Nondiabetic	Adjuvant chemotherapy and endocrine therapy ± Metformin	129	Phase 2	DFS: HR = 3.27, 95% CI [1.17–9.06] (*P* = 0.044)	Positive
	Zhao et al. (2017) [128]	Advanced/metastatic hormone receptor‐positive breast cancer	Aromatase inhibitor ± Metformin	60	Phase 2	mPFS: 4.7 vs. 6.0 months HR = 1.2, 95% CI [0.7–2.1] (*P* = 0.48)	Negative
	Nanni et al. (2018) [72]	Advanced metastatic HER2‐negative breast cancer Nondiabetic	Non pegylated liposomal doxorubicin + Cyclophosphamide ± Metformin	122	Phase 2	mPFS: 9.4 vs. 9.9 HR = 1.09, 95% CI [0.75–1.58] (*P* = 0.653)	Negative
	Roman et al. (2018) [129]	Neoadjuvant treatment breast cancer with metabolic syndrome	Chemotherapy ± Metformin	72	NA	Pathologic response tumor: 77.5% vs. 27.5% (*P* < 0.05)	Positive
	Pimentel et al. (2019) [130]	Advanced/metastatic breast cancer Nondiabetic	Chemotherapy ± Metformin	40	Phase 2	Mean PFS: 5.4 vs. 6.3 months, HR = 1.2 95% CI [0.63–2.31] (*P* = 0.58)	Negative
	Barakat et al. (2022) [71]	Neoadjuvant chemotherapy Nondiabetic	Chemotherapy ± Metformin	80	Phase 2	Pathological complete response: 22% vs 10% (*P* = 0.181) ORR: 80.6% vs. 68.4% (*P* = 0.236) Clinical complete response: 27.8% vs. 10.5% (*P* = 0.058)	Negative
	Essa et al. (2022) [131]	Advanced/metastatic Nondiabetic	Chemotherapy ± Metformin	107	Phase 2	Regression disease: 27.8% vs. 12.5% (*P* = 0.074) mPFS: 5 vs. 4 months (*P* = 0.753)	Negative
	Goodwin et al. (2022) [70]	Early breast cancer in adjuvant setting Nondiabetic	Standard of care ± Metformin	3649	Phase 3	DFS: HR = 1.01, 95% CI [0.84–1.21] (*P* = 0.93)	Negative
Hepatocellular carcinoma	El Shorbagy et al. (2020) [76]	Advanced/metastatic Diabetic and nondiabetic	Sorafenib ± Metformin	80	Phase 2	ORR: 52.5% vs. 55% (*P* = 0.5)	Negative
Melanoma	Montaudie et al. (2017) [75]	Advanced/metastatic	Metformin or placebo	17	NA	ORR: 0%	Negative
Ovarian	Hamedi et al. (2018) [132]	Localized ovarian cancer after cytoreductive surgery Nondiabetic	Chemotherapy ± Metformin	70	NA	4‐year rate of recurrence: 67.5% vs. 13.3% (*P* < 0.005)	Positive
	Zheng et al. (2019) [133]	First‐line chemotherapy at all stages Nondiabetic	Carboplatin + paclitaxel ± Metformin	47	NA	mPFS: 23 vs. 21 months (*P* = 0.68)	Negative
Colorectal cancer	Higurashi et al. (2016) [78]	Chemoprevention of colorectal cancer Nondiabetic	Metformin vs. placebo	151	Phase 3	Prevalence and number of adenomas or polyps after 1 year of treatment: 38% vs. 56.5%, RR = 0.67, 95% CI [0.47–0.97]	Positive
Pancreatic cancer	Kordes et al. (20150 [77]	Advanced/metastatic Diabetic and nondiabetic	Gemcitabine + erlotinib ± Metformin	121	Phase 2	OS at 6 months: 56.7% vs. 63.9% (*P* = 0.41)	Negative
	Reni et al. (2016) [134]	Advanced/metastatic Diabetic and nondiabetic	PEXG chemotherapy regimen ± Metformin	60	Phase 2	6‐month progression‐free survival: 42% vs. 52% (*P* = 0.61)	Negative

### 5.1 Breast cancer

In the MA.32 trial, 3643 nondiabetic patients with early breast cancer received randomized treatment with either metformin (850 mg daily) or a placebo for 5 years as adjuvant treatment. Metformin was used in combination with adjuvant chemotherapy, endocrine therapy, and trastuzumab as needed. More than 80% of the population required chemotherapy in addition to metformin, representing a high‐risk relapse group. The incidence of relapse was similar in both conditions, and the authors concluded that metformin did not confer a beneficial effect on disease progression [70]. In a neoadjuvant strategy, the outcome of 80 nondiabetic patients with locally advanced breast cancer was investigated in a randomized trial, where participants received neoadjuvant chemotherapy in combination with either metformin (1000 mg twice daily) or a placebo. Although there was a trend in the overall response rate, it was not statistically significant [71]. Finally, in a study at the metastatic stage, metformin was studied in combination with various cancer therapies. In association with anthracycline, 122 nondiabetic HER2‐negative metastatic breast cancer patients were randomized to receive the combination of nonpegylated liposomal doxorubicin, cyclophosphamide, and 2000 mg·day^−1^ metformin or placebo. The primary endpoint, progression‐free survival (PFS), was similar in both the groups after a median follow‐up of 39 months, with a median of 9 months [72].

### 5.2 Prostate cancer

Our laboratory conducted a phase II clinical trial in metastatic castration‐resistant prostate cancer patients. In this trial, metformin was randomized against placebo in 99 patients receiving docetaxel chemotherapy and androgen deprivation therapy. The PSA‐response rate reached 66% in both arms, without any significant difference in survival outcomes [73]. In contrast, the MANSMED trial that included 124 metastatic hormone‐sensitive prostate cancer patients demonstrated that metformin in combination with androgen deprivation therapy [74] improved the duration of progression to castration‐resistant prostate cancer without benefitting OS (29 vs. 20 months). MANSMED faced challenges due to the heterogeneous population of hormone‐sensitive prostate cancer patients at different stages of the disease. Factors such as presence or absence of metastatic disease and prior chemotherapy or locoregional treatment influenced the primary endpoint of progression to castration resistance, raising doubt on the significance of this outcome.

### 5.3 Melanoma

The use of metformin in third‐line treatment of melanoma did not improve survival. A 14‐center French study enrolled 17 patients with metastatic melanoma who had progressed after first‐line treatment (chemotherapy or BRAF inhibitor) and demonstrated ineligible for or lack of response to immunotherapy. Among these patients, 14 had received prior chemotherapy, and six had received ipilimumab and a PD‐1 inhibitor. At 6 months, none of the patients exhibited a partial or complete response [75].

### 5.4 Hepatocellular carcinoma and pancreatic cancer

In a study involving 80 patients with hepatocellular carcinoma, metformin (500 mg daily) was compared with placebo in combination with the VEGFR inhibitor sorafenib. However, no improvements in survival endpoints were observed. The only positive finding was that low plasma levels of VEGFR and HIF‐1α predicted a better response to the treatment [76]. In another study with 121 pancreatic cancer patients, metformin was randomized to placebo along with gemcitabine and erlotinib. The primary endpoint was OS at 6 months. Despite starting the metformin at 500 mg twice daily and subsequent increase to 1000 mg twice daily in the second week, OS at 6 months did not improve. However, they divided the population of 29 patients into two groups according to metformin plasmatic concentration. In an exploratory analysis, those in the high metformin concentration group (> 1.0 mg·L^−1^) had better survival than those in the low concentration group (9 vs. 6 months). Similar results were observed for patients whose insulin levels had been reduced by metformin. However, as this was a simple exploratory analysis, no definitive conclusions can be drawn from it due to the small number of participants [77].

### 5.5 Colon cancer

A phase III Japanese study of 500 patients was randomized to metformin vs. placebo for 1 year after colorectal adenomas or polyps resected by endoscopy. Metformin decreased the risk of polyps by 33%, but all polyps were either adenomas or hyperplastic polyps without colorectal cancer [78].

### 5.6 Lung cancer

In two trials involving advanced lung cancer treated with chemoradiotherapy, metformin was randomized, but both studies concluded that metformin did not exhibit antitumor activity [79, 80]. The Skinner study reported a progression rate at 1 year was 60% in the control group and 50% in the metformin group (HR 1.15 95% CI 0.77–1.73, *P* = 0.24). In Tsakiridis' study, metformin worsened survival, with a PFS at 1 year of 34% vs. 64%. Notably, the metformin group did not receive the prescribed doses of chemoradiotherapy and some doses of chemotherapy were omitted: 27% in the metformin group and 14% in the control group. This discrepancy was explained by high rates of severe toxicity (grade 3 or greater) in the metformin group (53% vs. 25%).

In a phase II trial, 2139 patients with advanced lung cancer were assigned to receive EGFR‐TKI (gefitinib and/or erdafinitib) or EGFR‐TKI plus metformin. The authors showed a significant improvement in PFS of 3 months and an increase of 14 months in OS. However, this study lacked randomization and stratification based on EGFR mutation profile and EGFR‐TKI treatment [81]. In addition, OS results may have been influenced by the 43% of the patients who switched to osimertinib, known for better results than gefitinib and/or erdafinitib according to the phase III FLAURA trial [82].

### 5.7 Metformin and immunotherapy

Currently, there are no published results from clinical trials combining metformin and immunotherapy. In a retrospective cohort of 55 patients treated with ipilimumab, nivolumab, and/or pembrolizumab, 22 patients were also treated with metformin. The objective response rate (68% vs. 54% *P* = 0.31), disease control rate (77% vs. 60%), median OS (46 vs. 28 months), and median PFS (19.8 vs. 5 months) were longer in the metformin cohort, although statistical significance was not reached, probably due to the small sample size [83]. This study raises the question of a potential synergistic anticancer effect between immunotherapy and mitochondrial inhibitors. Indeed, metformin has been shown to impact inflammatory genes, increasing the transcription of IL10 and IL1b in the late inflammatory response.

As clinical results are inconclusive at this stage, we await the outcomes of numerous ongoing clinical trials to make conclusions on metformin's antitumoral effects. In the meantime, several other inhibitors of OXPHOS have been synthetized. Here, we focus on IACS‐010759, the most studied mitochondrial inhibitor in recent literature.

## 6 IACS‐010759 targets OXPHOS vulnerability: *in vitro* and preclinical studies

**Fig. 3:**
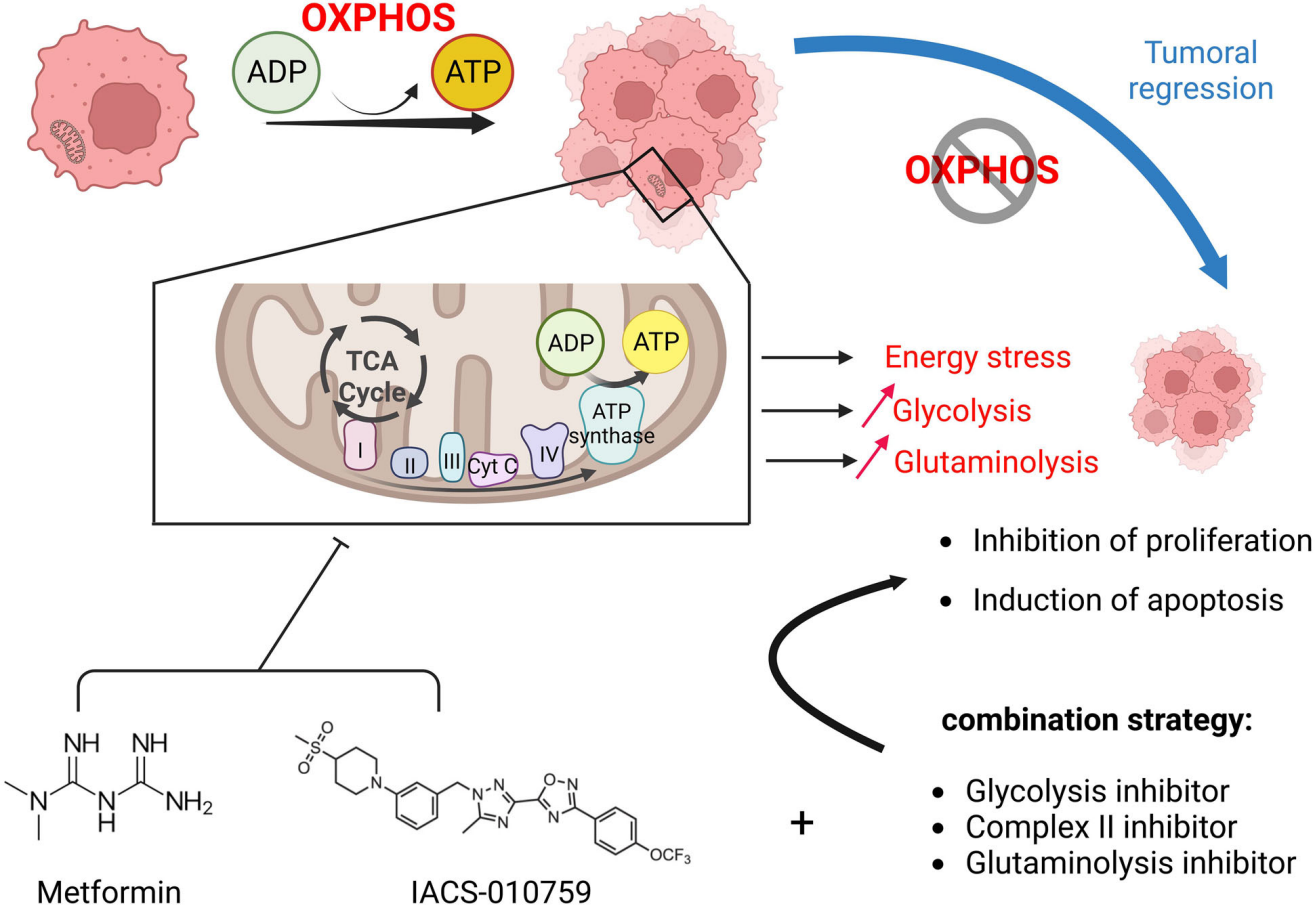
Metformin and IACS‐010759 target oxidative phosphorylation (OXPHOS) vulnerability. Cells that rely on OXPHOS for their energetic needs are sensitive to metformin and IACS‐010759. Both metabolic disruptors inhibit complex I of the electron transport chain (ETC), leading to major energy stress (decrease in adenosine triphosphate (ATP) concentration) and increased glycolysis and glutaminolysis. The combination with other metabolic inhibitors (glycolysis, complex II, or glutaminolysis inhibitors) leads to apoptosis and increased energy stress. (Figure done with Biorender).

IACS‐010759 is derived from BAY 87‐2243, a molecule identified via high‐throughput screening of a chemical library (~ 830 000 compounds) using a luciferase‐driven HIF‐1 reporter cell line in hypoxia [84]. Initially, BAY 87–2243 was found to inhibit HIF target genes and mitochondrial NADH–ubiquinone oxidoreductase (Complex I). Later, BAY 87‐2243 was demonstrated to reduce the viability of melanoma cells and tumor growth from BRAF mutant melanoma xenografts [85]. However, despite its great efficacy *in vitro* and in preclinical studies, the phase I clinical trial had to be stopped due to unexpected toxicities in humans. In response, structural modification of BAY 87‐2243 led to the synthesis of IACS‐010759 [8]. Despite these modifications, IACS‐010759 also exhibited adverse effects *in vivo* (see below). Molina et al. [8] evaluated IACS‐010759 in glycolytic‐deficient cells, including NB‐1 cells deficient for phosphoglycerate dehydrogenase (PGD) and D423 and Gli56 cell lines, which harbor a homozygous deletion for the glycolytic enzyme Enolase1 (ENO1). IACS‐010759 decreased viability and induced apoptosis in all tested cells, with ectopic expression of either ENO1 or PGD preventing the effect on viability. The same group assessed IACS‐010759's antitumor activity on mouse subcutaneous xenograft tumors from NB‐1 cells (human neuroblastoma) and observed strong tumor regression. These results illustrated the potent antitumor activity of IACS‐010759 in glycolysis‐deficient cells and emphasized the concept of OXPHOS vulnerability in cancers (Fig. 3).

To verify the specific targeting of Complex I by IACS‐010759, Seahorse assays were employed to measure the oxygen consumption rate (OCR) in detergent‐permeabilized cells. Treatment with IACS‐010759 in medium supplemented with pyruvate and malate resulted in a reduced OCR. However, OCR was not attenuated by IACS‐010759 treatment when medium was supplemented with succinate to fuel Complex II, thus overcoming complex I inhibition and showing the specificity of the compound [8]. The same group observed that the H292 clones, which harbor an amino acid substitution at Leu 55 (to Phe) in the ND1 subunit of Complex I, exhibited reduced susceptibility to IACS‐010759 [8]. Thus, they propose that IACS‐010759 binds to complex I next to the membrane‐embedded ND1 subunit. More specifically, to identify the binding position Tsuji et al. [86] used photoaffinity labeling experiments relying on a photoreactive IACS‐010759 derivate. Unlike other quinone‐site inhibitors, IACS‐010759 may interact with the middle of the ND1 subunit, which induces structural modifications of the quinone‐binding cavity, impacting the quinone redox reactions. This unique binding resulting in a specific inhibition of Complex I, which may be responsible for the cytotoxic properties of IACS‐010759 in cancer.

Leukemic cells and lymphomas are highly dependent on OXPHOS as circulating tumor cells must constantly adapt to face variations in nutrient and oxygen availability in the bone marrow microenvironment and blood circulation [87, 88, 89]. Regardless of the driving mutation, IACS‐010759 on its own or in combination triggers a cytotoxic response both *in vitro* and *in vivo*; for example, it induces apoptosis in models of acute myeloid leukemia (AML) [8, 90]. In synergy with AC220, an FMS‐like tyrosine kinase 3 inhibitor, a class III tyrosine kinase receptor expressed on the cell membranes of early hematopoietic progenitor cells, IACS‐010759 induces apoptosis in AML cells and synergistically reducing glucose and glutamine flux in the TCA cycle [91]. RNA sequencing (RNA‐Seq) analysis of T‐cell acute lymphoblastic leukemia (T‐ALL) cells with mutations in Notch receptor 1 (NOTCH1) identified enhanced OXPHOS and glutaminolysis signatures [92]. Glutaminolysis is considered a vulnerability in cancers since this pathway is crucial for anaplerosis, biosynthetic processes, and cellular redox purposes [93, 94]. Thus, Baran et al. [92] combined the inhibition of OXPHOS (via IACS‐010759) and glutaminolysis (via inhibitors of l‐asparaginase) and induced profound tumor reduction in preclinical models of T‐ALL. IACS‐010759, in synergy with an inhibitor of monocarboxylate transporter 1 (AZD3965), was shown to induce cell death in diffuse large B‐cell lymphoma [95]. Additionally, in mantle cell lymphoma, the combination of IACS‐010759 and ibrutinib (irreversible inhibitor of Bruton's tyrosine kinase) results in a cytotoxic response [96].

Studies conducted across various solid tumors have also identified OXPHOS vulnerability of these cancers. In lung cancer, mutations in the chromatin remodeling complex SWI/SNF, such as the genes SWI/SNF Related, matrix associated actin dependent regulator of chromatin, subfamily A, member 4 (*SMARCA4*) and AT‐rich interactive domain‐containing protein 1A (*ARID1A*), sensitize cells to OXPHOS metabolism. In this regard, IACS‐010759 treatment induced cell death in lung cancer cells harboring mutations in *SMARCA4* or *ARID1A* [97]. IACS‐010759 also improved the survival of mice with melanoma brain metastasis xenograft tumors [98]. Of note, high OXPHOS is associated with more oxidative stress. The transcription factor nuclear factor erythroid 2‐related factor 2 (NRF2) is a major regulator of antioxidant genes and a key component of the maintenance of intracellular redox homeostasis and the regulation of inflammation [99]. In head and neck squamous cell carcinomas (HNSCC) induced by human papillomavirus (HPV) infection, RNA‐Seq analysis revealed overexpression of NRF2 concomitant with an OXPHOS gene signature [100]. Hence, the authors hypothesized that HNSCC cells overexpressing NRF2 might exhibit vulnerability in OXPHOS. Consequently, they treated these cells with IACS‐010759. As anticipated, IACS‐010759 demonstrated significantly higher cytotoxic in a model of HNSCC cells overexpressing NRF2 compared with control cells [100].

Some studies suggest that IACS‐010759 could modulate immune cell activity, potentially leading to enhanced antitumor immune responses. Chen et al. [101] demonstrated that IACS‐010759, combined with radiotherapy, counteracts PD‐1‐resistant tumors in nonsmall cell lung cancer. In their conclusion, Fischer et al. [98] suggest that IACS‐010759 does not adversely affect CD8^+^ T‐cell function and could even be successfully combined with anti‐CTLA4 and/or anti‐PD‐1 therapies; however, this needs to be tested in melanoma models of brain metastasis. While the exact mechanisms by which IACS‐010759 affects the immune response still need to be elucidated, its potential to engage with immune checkpoints or immunosuppressive pathways raises the prospect of its integration into immunotherapeutic strategies.

In uveal melanoma, another study focused on tumors resistant to MEK inhibitor (MEKi) and tolerant to CDK4/6 inhibitor (CDK4/6i). RNA‐Seq analysis revealed an upregulation in the OXPHOS gene signature [102]. While MEKi, CDK4/6i, and IACS‐010759 alone did not induce significant apoptosis, the combination of the three triggered a significant apoptosis response. In high‐risk neuroblastoma, an increase in dihydrolipoamide S‐succinyltransferase (DLST, an enzyme that catalyzes the conversion of 2‐oxoglutarate to succinyl‐CoA) led to increased MYC levels, which rewires metabolism in favor of OXPHOS metabolism [103]. Combining the inhibition of both OXPHOS (via IACS‐01‐0759) and the TCA cycle resulted in striking synergy, inhibiting neuroblastoma cell growth.

Overall, inhibiting OXPHOS has been implicated in sensitizing apoptosis‐resistant cells in various types of cancers, such as platinum‐based chemoresistance in ovarian cancer [104] and resistance to ibrutinib in mantle cell lymphoma [105]. Inhibiting OXPHOS/ETC via IACS‐010759 and similar inhibitors, either in combination or alone, presents a promising strategy to overcome apoptosis resistance in cancer. Combination therapy offers several advantages, such as preventing resistance development, identifying drug synergies allowing decreased drug concentrations [106], and potentially reducing toxicity [107]. Repositioning existing drugs in a new therapeutic context may provide new possibilities for reduced cost and time development [108]. A recent study illustrated how targeting OXPHOS via IACS‐010759 treatment sensitizes AML and multiple myeloma (MM) to venetoclax‐induced apoptosis [109, 110]. Venetoclax (a BH3 mimetic), a Bcl‐2 antagonist used to trigger apoptosis in cancers, inhibits proliferation and colony formation in AML [109], and in combination with venetoclax induces strong apoptosis in both AML and MM. The inhibition of the ETC via IACS‐010759 treatment increases apoptosis‐regulating proteins such as the transcription factor activating transcription Factor 4 (ATF4) and the proapoptotic protein NOXA, potentially increasing Bcl‐2 availability and interaction with venetoclax [110]. Additionally, in combination with vinorelbine (a microtubule dynamics inhibitor), IACS‐010759 decreases the viability of AML cells [111]. More recently, El‐Botty et al. [112] showed that IACS‐010759 inhibits the growth of estrogen receptor‐positive breast cancer patient‐derived xenograft resistant to palbociclib (a CDK4/6 inhibitor). This study clearly shows that tumors with high OXPHOS are a potential reliable target for IACS‐010759.

Altogether, *in vitro* and preclinical studies strongly support the use of IACS‐010759 in humans. Metformin, being directly available, holds the for phase II clinical trials, while IACS‐010759 is a potent and pharmacologically active molecule against energy metabolism.

## 7 Lessons from the clinical trial with IACS‐010759

IACS‐010759 has been tested in its first‐in‐human study (NCT02882321) involving AML and solid tumor populations [113]. The study enrolled 17 patients with relapsed/refractory AML and 23 patients with a solid tumor. The primary objectives were to determine the safety, tolerability, and maximum tolerated dose, while the secondary objectives evaluated the pharmacokinetics and antitumor activity of IACS‐010759.

A dose escalation of IACS‐010759 was performed for AML and solid tumors. Elevated blood lactate and neurotoxicity were the most common adverse events (AEs) and contributed to the early termination of the study. Grade 3 or higher adverse events included increased blood lactate levels in 53% of AML patients and 9% of solid tumor patients, lactic acidosis in 24% of AML patients, and peripheral neuropathy (weakness and paresthesia of the hand and/or feet and/or leg/hip, but no painful neuropathy) in 6% of AML patients and 4% of solid tumor patients. Nerve biopsies from two solid tumor patients confirmed the diagnosis of mixed axonal and demyelinating neuropathy; however, no toxic deaths were observed. According to the clinical and biological progression criteria for AML (European LeukemiaNet 2017) and radiological criteria (RECISTv1.1 criteria) for solid tumors, the best objective response rate for IACS‐01059 was a partial response in one patient with castration‐resistant prostate cancer and stable disease in eight other solid tumors. As with all phase I studies, survival data were not reported.

In conclusion, a significant challenge with IACS‐01059 is to concomitantly maintain the minimum plasma concentration at 20 nm (as defined by preclinical studies), to achieve an antitumoral effect while mitigating dose‐dependent adverse effects. Similar to other OXPHOS inhibitors, IACS‐010759 induces a metabolic switch toward glycolysis, resulting in an increased plasmatic concentration of lactate. Elevated lactate induces inflammation‐driven pain, including that associated with neuropathy. A better understanding of the effects of IACS‐01059 on normal tissue and, particularly on neurons, will help in improving its tolerability and, consequently, its overall efficacy.

## 8 Metformin vs. IACS‐01059: strengths and drawbacks

There is a notable discrepancy between experimental preclinical studies and prospective clinical studies for both drugs. Retrospective observational studies, solely performed with metformin, yielded highly encouraging results but are in contradiction with the results of the clinical trials. One explanation proposed by Yu and Suissa [114] points to time‐related bias: in observational studies, metformin and other antidiabetic drugs, such as sulfonylurea, might have been prescribed simultaneously. However, patients who were first prescribed a sulfonylurea treatment and then metformin, were classified as ‘metformin users’. The period between initiating sulfonylurea treatment at the cohort's outset and the first prescription of metformin is called the ‘immortal time’, potentially artificially prolonging the survival duration of metformin users. For both drugs, the lack of effects in patients can be explained by the fact that most of the clinical trials recruited patients with advanced metastatic cancers resistant to treatments.

To our knowledge, there are currently no ongoing clinical trials testing IACS‐01059 in preventive studies, whereas some protocols are recruiting patients to study the effects of metformin on cancer incidence. The Metformin in Li Fraumeni (MILI) trial is designed to study the preventive effects of metformin in patients with Li‐Fraumeni syndrome, an inherited disorder caused by TP53 mutation, leading to the development of a wide range of cancers (brain, blood, soft tissues). The MILI study aims to recruit 200 patients in the UK who will be randomly treated with metformin or not. In prostate cancer, the UK STAMPEDE trial, a multiarm, multistage (MAMS) randomized controlled trial, enrolled almost 12 000 patients since 2005. The target population is men with high‐risk localized or metastatic prostate cancer. STAMPEDE had already reported practice‐changing results, such as adding docetaxel or abiraterone to improve disease control and life expectancy or adding radiotherapy for low‐volume metastatic prostate cancer. The control arm was arm A with standard of care (SOC), which may evolve based on scientific evidence. Currently, the SOC is androgen deprivation therapy with docetaxel for high‐risk metastatic prostate cancer and radiotherapy for low‐risk prostate cancer and locally advanced prostate cancer. In Arm K, STAMPEDE will investigate whether adding metformin to the current standard of care for nondiabetic men can improve survival [115].

Metformin was well‐tolerated in cancer clinical trials, with moderate low‐grade adverse effects such as diarrhea and nausea. However, more severe side effects were observed with IACS‐01059, including elevated blood lactate, peripheral neuropathy, and vomiting leading to treatment discontinuation in some patients [113]. Increased lactate concentration is frequent in preclinical model following treatment with OXPHOS inhibitors including metformin [85, 116]. Notably, the dose administrated to animal models in these experiments is at least twice higher than the dosage for humans. Plasmatic dosage of metformin was performed in few clinical trials, Kordes et al. observed a better response for patients with high concentration (equivalent to 7.7 μm) whereas for IACS‐01059, a minimum of 20 nm is required to have an antitumoral effect. The discrepancy between the two compounds may reside in the fact that IACS‐01059 is a more potent inhibitor of complex I and has a better bioavailability than metformin [8]. Indeed, metformin is highly polar and requires specific transporters to enter the cells, namely, organic cation transporters (OCTs) [117]. Thus, the responsiveness of tumor cells to drugs depends on the expression of OCTs. Furthermore, metformin is a weak inhibitor of complex I [118] with an IC_50_ value in the millimolar range vs. nanomolar for IACS‐01059. Thus, the efficacy of metformin as a complex I inhibitor *in vivo* remains uncertain due to the high concentration required *in vitro*.

## 9 Conclusion

The promising outcomes seen in numerous experimental studies involving metformin and IACS‐01059 raised hope for their potential in cancer therapy. However, the subsequent lack of efficacy and adverse effects tempered our expectations. Despite these setbacks, the aim to find new tools to target energy metabolism remains crucial. Key considerations in this regard include: (a) prioritizing the search for new molecules with improved specificity and reduced toxicity, such as CPI‐613 or CRO‐15 [119, 120], both potential candidate drugs for future clinical trials. It is essential to note the well‐tolerated nature of CPI‐613 in leukemic patients [121]; (b) designing new drugs not only to target cancer cells but also immune cells to enhance the antitumoral response. To address this, testing the efficacy of new mitochondrial inhibitors on both cancer and immune cells of the TME, under coculture conditions, becomes vital. Patient‐derived tumoroids offer unique opportunities for personalized therapy and a deeper understanding of the complex effects of metabolic disruptors on cancer and immune cells. Although the journey ahead is challenging, the ‘one stone, two birds’ strategy could reinvigorate antimitochondrial drugs.

## Conflict of interest

The authors declare no conflict of interest.

## Author contributions

MPM, AB, MK, PP, MR, NMM and FB wrote the paper. SB, NMM and FB did the illustrations. [Correction added on 03 April 2024, after first online publication: The Author contributions section has been included in this version.]
